# Human–machine cooperation meta-model for clinical diagnosis by adaptation to human expert’s diagnostic characteristics

**DOI:** 10.1038/s41598-023-43291-8

**Published:** 2023-09-27

**Authors:** Hae-Jeong Park, Sung Huhn Kim, Jae Young Choi, Dongchul Cha

**Affiliations:** 1 Department of Nuclear Medicine, Department of Psychiatry, Graduate School of Medical Science, Brain Korea 21 Project, Yonsei University College of Medicine, Seoul, South Korea; 2 Department of Cognitive Science, Yonsei University, Seoul, Republic of Korea; 3 Center for Systems and Translational Brain Sciences, Institute of Human Complexity and Systems Science, Yonsei University, 50-1, Yonsei-ro, Sinchon-dong, Seodaemun-gu, Seoul, 03722 Republic of Korea; 4 Department of Otorhinolaryngology, Yonsei University College of Medicine, Seoul, South Korea; 5 Center for Innovative Medicine, Healthcare Lab, NAVER Corporation, 95, Jeongjail-ro, Bundang-gu, Seongnam-si, Gyeonggi-do 13561 Republic of Korea; 6 Healthcare Lab, Naver Cloud Corporation, Seongnam-si, Republic of Korea

**Keywords:** Computational biology and bioinformatics, Machine learning, Medical imaging

## Abstract

Artificial intelligence (AI) using deep learning approaches the capabilities of human experts in medical image diagnosis. However, due to liability issues in medical decisions, AI is often relegated to an assistant role. Based on this responsibility constraint, the effective use of AI to assist human intelligence in real-world clinics remains a challenge. Given the significant inter-individual variations in clinical decisions among physicians based on their expertise, AI needs to adapt to individual experts, complementing weaknesses and enhancing strengths. For this adaptation, AI should not only acquire domain knowledge but also understand the specific human experts it assists. This study introduces a meta-model for human–machine cooperation that first evaluates each expert’s class-specific diagnostic tendencies using conditional probability, based on which the meta-model adjusts the AI’s predictions. This meta-model was applied to ear disease diagnosis using otoendoscopy, highlighting improved performance when incorporating individual diagnostic characteristics, even with limited evaluation data. The highest accuracy was achieved by combining each expert’s conditional probabilities with machine classification probability, using optimal weights specific to each individual’s overall classification accuracy. This tailored model aims to mitigate potential misjudgments due to psychological effects caused by machine suggestions and to capitalize on the unique expertise of individual clinicians.

## Introduction

Deep learning-based diagnostic assistant systems have made significant strides in various medical fields, such as radiology, retinal fundus, and dermatology images^1–10^. While these systems have demonstrated performance comparable to domain specialists, the integration of these technologies into real-world clinical practice remains an underexplored challenge. One pivotal concern surrounding these systems is liability. Hence, assistive cooperation of artificial intelligence (AI) to human intelligence (HI) is imperative^10^. This cooperation means AI aids in the decision-making process, with human experts retaining final decision-making responsibility. A few studies have focused on incorporating DL models to help medical experts in various tasks^10–12^.

The differences between training AI models and human experts and their distinct strengths and limitations in the diagnostic process lead to AI-HI cooperation. For instance, AI models have shown statistical bias towards prevalent diseases^13,14^, yet maintain remarkable consistency^15^. Conversely, human experts, while not as biased towards prevalent conditions, exhibit significant inter-rater variability^16,17^. This variability can sometimes prompt patients to seek multiple opinions. These distinctions should be considered in the cooperation between humans and machines for practical use.

In a real-world setting, automated diagnostic systems primarily serve as assistants, offering insights from AI as a supplementary opinion. However, if a diagnostic assistant system makes a suggestion based solely on the outcome of AI classification, users, especially less experienced physicians, could be biased toward or against the suggestion of the AI^10^. A promising alternative is a cooperative model that treats both humans and AI as independent classifiers and merges their outputs. However, due to the substantial variability in human experts’ accuracies and diagnostic tendencies, such a unified model might exhibit unpredictable performance for experts with different expertise in clinical practice.

Thus, the ideal AI system should be tailored to individual practitioners, accounting for their unique diagnostic strengths and biases. Such adaptability not only harnesses the domain-specific expertise of the human but also aligns with their diagnostic tendencies. This alignment is vital; experts’ skepticism towards AI decisions, especially when they conflict with their own judgments, might lead to the rejection of correct AI suggestions even in cases the human expert is not proficient. Adapting AI to individual human diagnostic characteristics might mitigate such challenges, ensuring smoother AI-HI collaboration. Therefore, AI should learn not only the domain knowledge but also the human partners’ tendencies that it assists.

In this study, we advocate for an AI-HI cooperation strategy tailored to individual diagnostic traits. By first assessing an expert’s proficiency with a small set of labeled data, we formulate a meta-model to maximize each party’s strength in integrative diagnosis by adaptively weighting each player’s diagnostic characteristic for the final decision. We compared this AI-HI meta model with a simple ensemble method that adds each party’s predictions according to predefined weight. By doing so, we argue that the cooperation of human and machine experts should be individual-specific, considering the pros and cons of each human expert for clinical usage.

Using ear disease classification from otoendoscopic images—which shows heterogeneous diagnostic performance among human experts^15^—we underscore the practicality of this cooperative strategy. Ear and mastoid diseases are common in, but not limited to, developing countries^18^. They are one of the core medical licensing exam tests, and primary care physicians are expected to treat these disease groups. However, medical students may not receive sufficient training on otoscopy^19^, and even for experts, the performance may not be good enough to ensure consistency^20^. By testing our meta-model with a diverse group of physicians, including six otolaryngologists or otolaryngology residents and eight non-otolaryngologists, we seek to address these challenges.

## Results

This study used convolutional neural network (CNN)-based otoendoscopy classification models (see Cha et al.^15^) trained with 6,900 otoendoscopic images of six classes (Table 1). Table 1 summarizes two independent data sets used to train and test the cooperation models. The first test set (Test 1) is equally distributed, while the second test set (Test 2) is chosen according to the imbalanced prevalence. Six ENT (Ear, Nose, and Throat) physicians and eight non-ENT physicians (family medicine specialists, emergency medicine specialists, and general practitioners) scored an average ± SD of 71.17 ± 3.37% and 45.63 ± 7.89%, respectively. Our ensemble AI model, based on pre-trained DL models (ResNet152, DPN92, Inception-V4, and Densenet201) and modified to handle the class imbalance problem, scored an accuracy of 80.33% in balanced test set (Test 1) and 86.67% in imbalanced test set (Test 2). For detailed results, see Cha et al.^15^. We compared the proposed method with a simple ensemble method in combining human experts and AI’s decisions.

**Table 1:** Training and test sets for otoendoscopic images.

Classification	Number of images		
	Training (%)	Test 1 (%)	Test 2 (%)
(1) Tympanic perforation	1793 (25.99)	50 (16.77)	51 (17.00)
(2) Attic retraction/Atelectasis	521 (7.56)	50 (16.77)	20 (6.67)
(3) Myringitis/Otitis externa	256 (3.71)	50 (16.77)	15 (5.00)
(4) Otitis media with effusion	506 (7.33)	50 (16.77)	29 (9.67)
(5) Tumors	285 (4.13)	50 (16.77)	18 (6.00)
(6) Normal	3539 (51.29)	50 (16.77)	167 (55.67)
Total	6900 (100.0)	300	300

### Synergistic effects of AI and human experts according to human expertise and classes with different prevalences

**Figure 1:**
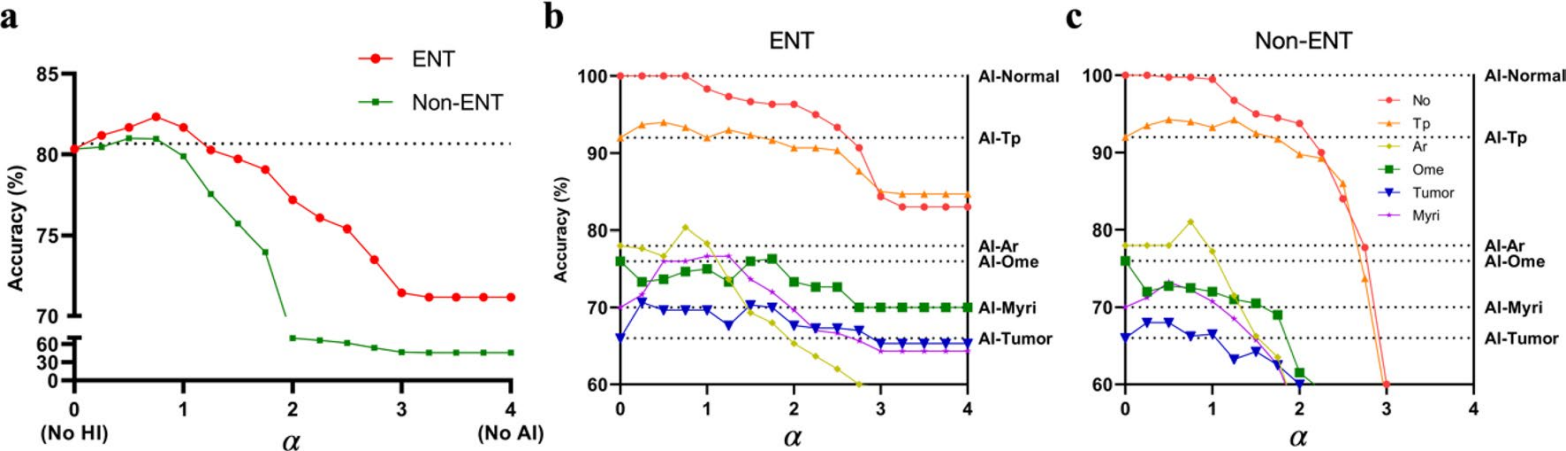
A human weight (α) and its overall accuracy for analysis of human–machine cooperation. The alpha value (α) indicates the weight of the HI decision. Since the model consists of humans and four different AI models, the alpha value ranged from 0 (no human intelligence) to 4 (no artificial intelligence). (**a**) Otolaryngologists improved the overall accuracy, while non-otolaryngologists have limited contributions. The dashed grid line indicates the overall accuracy of the ensemble DL model (α = 0), which is 80.33%. Peak accuracy was 82.33% (2.43% increase) in ENT (α = 0.75) and 81.00% (0.09% increase) in non-ENT (α = 0.5). The overall average accuracy of each group (α = 4) is 71.17% and 45.63% in ENT and non-ENT physicians, respectively. The mean recall for each class was displayed in the ENT group (**b**) and the non-ENT group (**c**). When appropriately counted, physicians’ diagnostic result enhances the diagnosis of rare cases (e.g., Myri and Tumor). No, normal; Tp, tympanic perforation; Ar, Attic retraction; Ome, otitis media with effusion; Myri, myringitis (otitis externa); Tumor, middle or external auditory canal tumors, or cerumen impactions. Dotted horizontal lines indicate the reference recall of AI for each class.

To evaluate the synergistic effects of human experts and DL models according to classes, we tested the performance of a simple ensemble between HI and AI by weighting the top 4 performing DL models’ softmax values and a human rater’s diagnosis. Since otolaryngologists were often better at predicting less common disease entities, appropriately combining decisions from both parties synergized overall accuracy (Fig. 1a). This was even true for non-otolaryngologists. Still, the benefit was minimal since the diagnostic performance was far worse than DL models. We also assessed the per-class accuracy of six individual classes.

Each class had a different optimal human weight value: smaller classes, such as tumors and myringitis, had higher human weight values, which implies better performance in the human–computer hybrid classifier than machines alone in these classes. Since DL models outperformed both human physician groups for each class, setting the alpha (human weight) value more than 3 had an adverse effect on class-specific outcomes in otolaryngologists (Fig. 1b). In non-otolaryngologists, accuracy was usually better off by allowing less physician’s input to reflect the final result (Fig. 1c).

This experimental result can be summarized in two points: (1) an appropriate combination of both human experts and artificial intelligence synergizes overall accuracy, and (2) the class-specific tendency of each individual might play a more critical role in practical human–machine cooperation than merely weighting each individual’s general accuracy. Thus, we took these two points as the core for the current meta-model.

### AI and HI: cooperation meta-models

The experimental AI-HI cooperative classification models were designed to blend the human and DL models’ classification results with appropriate weighting. The proposed cooperative model first incorporated the overall accuracy of individuals: weighting high for experts with higher performance. The cooperative classifier then weighted the human rater’s class-specific diagnostic characteristics and the DL models’ predictions. A human rater’s diagnostic characteristics are assessed from the confusion matrix of the human expert for an evaluation data set in the form of the conditional probability of being a true class for a diagnosed class by an individual.

As described in the methods section, we sampled each clinician’s given answer and correctness from balanced test set (Test 1 dataset) from zero up to 200 samples. We compared the proposed AI-HI cooperation model of the performance-weighted conditional probability of individual (PCoptMH) with (1) a simple average of the conditional probability of the human diagnosis and machine classification probability (CavgMH) and (2) the optimally weighted average of the human delta response (1 for the diagnosed class, and 0 for the other classes) of an individual expert and machine classification probability (PoptMH). The PCoptMH considers each human expert's overall diagnostic performance and class-specific diagnostic tendency. Meanwhile, the CavgMH considers the class-specific diagnostic tendency but does not consider each individual’s overall performance: it does not differentiate highly accurate human experts from those with low accuracy. In contrast, the PoptMH considers each human expert's overall performance but does not consider class-specific diagnostic characteristics.

**Figure 2:**
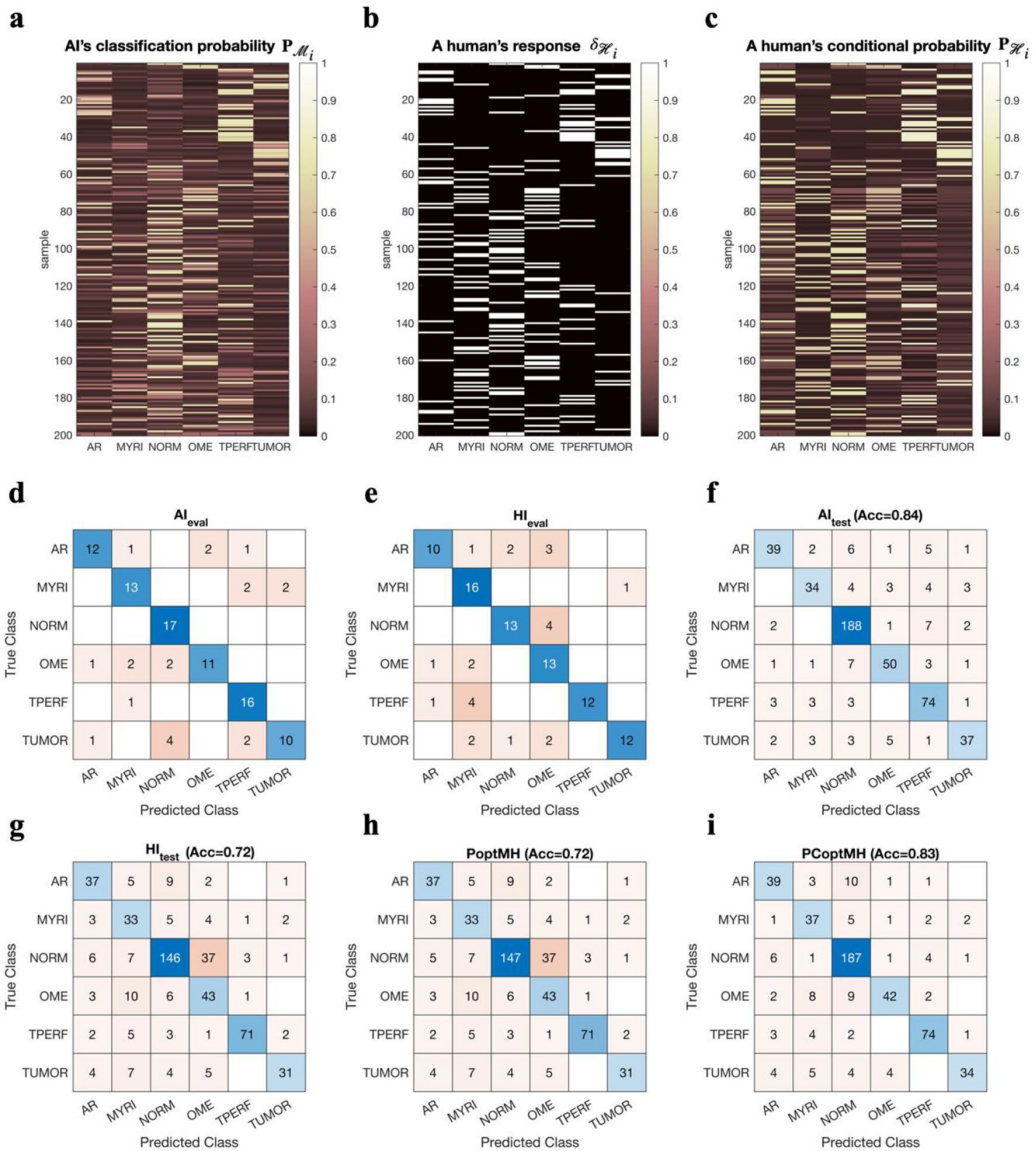
A demonstration of the AI-HI cooperation scheme. Cooperation between AI models and a human expert (HI, an ENT resident, 3rd year) is presented. (**a**) The DL classifier generates outputs corresponding to the probability of being each class resulting from the softmax layer. (**b**) Human experts choose a class among six classes, represented as a delta-response vector assigning one to the diagnosed class, and the other classes are set to zero. (**c**) We used a conditional probability of a true label for a given human diagnosis label to consider the human expert’s classification tendency, i.e., accuracy for each class. The conditional probability of a true label is derived from one’s confusion matrix of the evaluation data set. (**d**) Confusion matrix of AI and (**e**) confusion matrix of a human otolaryngologist (HI) for the evaluation data (100 samples of Test 1), (**f**) confusion matrix of AI predictions, and (**g**) confusion matrix of a human expert for the test dataset (500 samples of Test 2 and Test 1 except for the 100 samples for evaluation) are displayed. (**h**) Confusion matrix of a cooperation model between AI and HI by optimizing weight for AI predictions in (**a**) and HI delta-response in (**b**) according to HI’s overall accuracy (PoptMH), without considering individual characteristics for classes in each person, for the test dataset and (**i**) confusion matrix of the cooperation model optimally adding AI predictions in (**a**) and HI conditional probability in (**c**) by considering overall personal accuracy and diagnostic pattern of classes of human expert (PCoptMH) for the test dataset are displayed.

Figure 2 shows an example of this cooperative procedure applied to a third-year otolaryngology resident. From the classification results and diagnosis results for 100 evaluation samples of Test 1 dataset, we evaluated the DL models’ and the physician’s overall accuracy and class-specific tendency (Fig. 2a, b). Then, we built a conditional probability of the clinician (Fig. 2c) based on the confusion matrix of the clinician for the evaluation data set (Fig. 2d, e).

For comparison purposes, the accuracies of the DL model and the human expert are depicted in the confusion matrix using a new test set (Fig. 2f, g). This test set comprised both Test 1 and Test 2 datasets, excluding the 100 evaluation samples selected from the Test 1 dataset, resulting in 500 samples. This arrangement ensured that all diagnostic labels rated by each individual were included. The same test set was also employed to assess the performance of the cooperation methods, PoptMH and PCoptMH (Fig. 2h, i). In subsequent analyses involving different evaluation samples, the test dataset was assembled following the same method.

Using cooperation with PoptMH resulted in only a minimal gain in accuracy (Fig. 2h). Applying the PCoptMH, a slight decrease in overall accuracy was obtained compared to DL models alone (0.84 vs. 0.83, Fig. 2f, i); however, the decision was more tailored toward the physician’s decision, which respects individual tendencies to diagnose each class.

**Figure 3:**
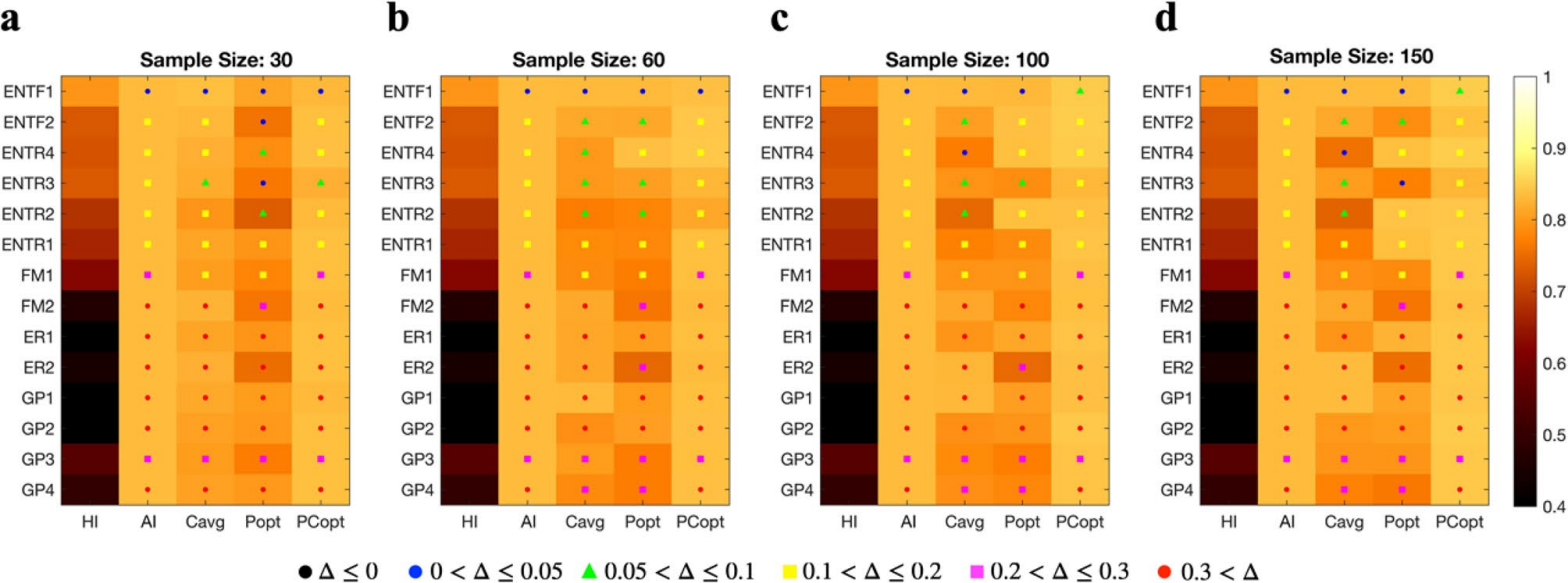
The performances of human–machine cooperation models according to evaluation sample sizes. (**a**)–(**d**) Displays the performance of human experts (HI), DL model (AI), CavgMH, PoptMH, and PCoptMH for probing samples of 30, 60, 100, and 150 to evaluate the human expert’s characteristics. The CavgMH is a simple averaging method between DL classifier probability and the conditional probability of human ratings without optimizing weights for each individual’s performance. PoptMH indicates AI-HI cooperation with optimized weight for the person’s contribution to the final decision in averaging the DL classifier probability and human delta response (1 for the diagnosed class, 0 for others) without using the conditional probability of true labels for given human ratings. PCoptMH indicates AI-HI cooperation with optimized weight for the person in adding the DL classifier probability and the conditional probability of human decision. The difference Δ (gain) between HI and the other four models is displayed. Blue dots indicate 0 < Δ ≤ 0.05, green triangle, 0.05 < Δ ≤ 0.1, yellow square, 0.1 < Δ ≤ 0.2, magenta square, 0.2 < Δ ≤ 0.3 and red dots, 0.3 < Δ. Black dots indicate a decrease in accuracy. Although the DL model generally shows higher performance than human experts and cooperation may lose performance that the DL model alone has in some experts, this approach takes account of the human’s knowledge and responsibility. Overall accuracy was gainable across almost all individuals, even when using small samples of evaluating human rater’s skills. ENTF, otolaryngologists; ENTR, otolaryngology residents (numbers indicate years of training); FM, family medicine specialists; ER, emergency medicine specialists; GP, general practitioners.

In summary, increased accuracy was obtained across almost all groups when there were sufficient data for evaluating human rater skills—by adding machine classification probability and the conditional probability of each individual’s classification (Fig. 3, CAvgMH). Combining the conditional probability of human decisions for each class with overall personal accuracy (PCoptMH) produced more accurate results than only using each human expert's overall accuracy (PoptMH).

Regarding practical points, the performance did not require many probing samples to estimate the confusion matrix for each person. Even five samples for each class (30 in total, Fig. 3a) helped capture the tendency of human classification and improve the cooperative classifier.

## Discussion

DL models are expected to play a cooperative role in clinics^3,21^ with human intelligence because the two have different diagnostic strategies and may complement each other. In our previous study^15^, DL models show consistent performance but weakness in imbalanced data, as indicated by higher prediction bias toward prevalent cases. In contrast, human raters showed higher individual variances but little bias toward prevalence^15^. We speculate this is due to differential learning or training tactics between humans and computers.

Collective intelligence (CI) was introduced in medical diagnostics^22^ and decision-making^23^ and proved beneficial. In a study^22^ with skin cancer detection, diagnostic accuracy should be similar between doctors to enhance the overall detection accuracy. In another study^23^, which focused on mammography screening, CI increased true positives and decreased false positives and helped overcome the decision accuracy of a single radiologist.

Our study can be viewed as CI between humans and computers, as our experimental model independently takes input from both parties. A similar approach has been studied in skin cancer classification^24^, predicting the course of multiple sclerosis^25^. In the skin cancer classification by Hekler et al.^24^, they classified dermoscopic images into five categories and combined dermatologists’ answers, which were independently taken. They applied the XGBoost^26^ algorithm to combine the probability of each class label with the dermatologist’s answer. In another study, they surveyed medical students to predict the duration of the relapsing–remitting phase in multiple sclerosis. They used random forests^27^ to train the DL model and bootstrapping with medical students’ predictions to enhance the classification performance. Of note, they also used a linear combination of predictions of humans and computers but got worse performance.

Weighted averaging between DL models and human physicians showed improvement in diagnostic accuracy, especially in minor classes (Fig. 1). As an exception, otitis media with effusions were hard to diagnose because of many subtle cases, and physicians were better off by accepting the DL model’s answers. But for tumors and myringitis classes, human physicians showed the possibility of aiding the DL model’s lack of data availability. In non-otolaryngologists, since the diagnostic skills are too inferior to DL models, they could offer little help to increase the accuracy.

With the conditional probability method, our system analyzes the strengths and weaknesses of the human experts and weighs DL results to make suggestions depending on the situation: it provides strong suggestions when DL is superior and weak suggestions when DL is vulnerable. As shown in Fig. 2, PoptMH utilizes the delta decision of each individual (1 or 0 for classes) when combined with the machine classification probability. In contrast, PCoptMH uses conditional probabilities representing an individual’s diagnostic tendency. By introducing the conditional probability of a diagnosis of each human expert, cooperation performance is increased compared to simple weighting for the human expert’s overall accuracy. In this manner, the combined prediction is tailored to the specific physician.

It should be noted that our proposed system does not require an extensive number of samples to evaluate each human expert’s classification performance or bias. Only 30 or 60 samples are sufficient to learn human experts’ diagnostic characteristics. This makes the current method suitable for real-world clinics, as shown in Fig. 3.

The relative merits of the parallel cooperation of humans and machines and the sequential approach from the machine to the human (the DL model provides an opinion to the human expert) have been argued. The sequential approach may be a conventional concept for AI–HI cooperation in medicine. However, in a previous study^10^, the diagnoses made by human raters were adversely affected when the computer made faulty suggestions, especially in less experienced physicians. The strength of our system is that it takes predictions by humans and the DL model as inputs independently. Therefore, users may avoid the psychological effects initiated by the suggestions of the prediction systems. Parallel cooperation, however, could be of disadvantage in terms of time-saving since it requires human physicians’ answers to see the result of the meta-model. However, inspecting eardrums and external auditory canals does not usually require extensive time as to radiologic image analysis. Therefore, in this situation, the parallel cooperation in the current study makes a plausible choice.

Although the accuracy of the predictions of the AI model exceeds most human raters, relying on automated systems without human guidance is discouraged. As noted in a study on skin cancer^28^, physicians put clinical information together with imaging information, which may ultimately result in a more accurate diagnosis than considering image alone as in AI models. Also, the current DL model treats all diseases equally. The system does not know that it is dire to misdiagnose diseases such as external auditory canal cancer.

We can extend the current parallel cooperation to serial cooperation, i.e., AI decision result is suggested to a human expert. Instead of directly providing the classification decision in terms of the final class label or the softmax probability of the classifier, we may adjust the suggested probability by weighting the classification probability of the machine with the inverse of human conditional probability (for the false classes) to guide the human decision. An experiment with this scheme will be further explored.

Diagnosing otologic diseases from a single image poses significant challenges, even for physicians with extensive experience^20^. Physicians may lack confidence in diagnosing certain diseases, especially when dealing with complex ear pathologies unless they have received specialized training. This is where DL models can be particularly beneficial, aiding physicians by not only enhancing overall accuracy but also instilling confidence in their diagnostic decisions, akin to consulting with a colleague. Nevertheless, the dynamics of an individual’s confidence in the machine, as well as in their own judgments, warrant further exploration in future studies.

The current study has some limitations. Otoendoscopic images often contain more than one finding, which calls for multi-label multi-class models in future studies for a more realistic automated diagnosis system. In this study, if more than one pathology was present, labeling was performed according to a predefined labeling priority, but at the time of prediction, only one class was chosen as the argmax value, not the labeling priority, due to the AI model’s design. In addition, we presented a cooperation model only for the case of otoendoscopic image classification. The cooperation model should be validated in other domains of cooperation between human experts and machines. Also, even though we obtained parallel input from both parties, the final confirmation should be done by the attending physician because of the reasons mentioned earlier. This is eventually double consideration of the human rater. Most importantly, the design of the cooperative classifier was post-hoc, which is based on snapshots of physicians’ answers. Physicians’ decision accuracy is likely to increase as they gain more experience, whether with our diagnostic assistance or not. Future studies on the collaborative model should be designed to follow physicians’ enhancement of skills so that the model could rely more on human physicians’ answers. Also, DL models with consideration of clinical information as well as images, which are multi-modal systems, should be conducted to offer a more physician-like model in the future.

In conclusion, we suggest a cooperation method that weighs the strengths and weaknesses of both parties for improved and consistent healthcare services. For this, the system first assesses the diagnostic characteristics of human experts for all classes. Based on this individualized assessment, the proposed model appropriately respects both the user diagnosis patterns and DL models by independently taking answers from both parties. Furthermore, the model minimizes psychological effects often present in conventional diagnosis assistant systems. We did not use domain-specific knowledge in the AI-HI cooperation meta-model; hence, the strategies we applied are not confined to otoendoscopic image classification. It may be generalizable to all decision-making tasks, where individual human knowledge plays an important role.

## Materials and methods

### AI classifiers

We used CNN-based DL models for otoendoscopy classification, which we previously reported in Cha et al.^15^. The DL models were trained with 6900 images out of 7500 otoendoscopic images of patients who visited the outpatient clinic in a tertiary referral center (Severance Hospital, Seoul, South Korea, Department of otorhinolaryngology). The details of labeling and training of the models can be referred to Cha et al.^15^.

In brief, otoendoscopic photos of the tympanic membrane and the external auditory canal (EAC) were labeled into six categories based on the *Color Atlas of Endo-Otoscopy*^29^ (Table 1). If more than one etiologies were present in the image, it was labeled according to our labeling order, determined by the clarity of the diagnosis and the next required step in real-world clinics. Post-surgery status, similar images, including the same patient’s eardrum image from multiple angles, blurry images, and otoendoscopic images from the same patient’s follow-up data, were excluded. To minimize noisy labeling, three additional steps were taken. First, we checked the medical record of the given image created by the attending physician at the time, who had at least ten years of clinical experience in our center. Second, we also checked audiometric and radiologic test results when the otoendoscopic image could not be classified clearly. Lastly, the image was excluded if the last author (D.C.) could not agree even after the aforementioned steps.

ImageNet pre-trained CNN models were used to perform transfer-learning of otoendoscopic images. After several models, four top performers were chosen for further optimization: ResNet152^30^, InceptionV4^31^, DPN92^32^, and DenseNet201^33^. Affine transformations on images (horizontal flip, rotation, random scales, levels, and warping) were performed when augmenting otoendoscopic image data when oversampling. Since the training dataset was imbalanced, we used oversampling, mixup^34^, and focal loss^35^ (γ = 1) to mitigate the class imbalance problem. Training, validation, and testing were implemented using Pytorch with Fastai library^36^.

### Participants and experiments

A computerized online questionnaire consisting of two each mutually exclusive sets containing 300 anonymized otoendoscopic images (Table 1) was presented to fourteen physicians: six otolaryngology department personnel (two otolaryngologists, four otolaryngology residents) and eight non-otolaryngologist but practicing otoscopy in clinics: two emergency medicine specialists, two family medicine specialists, and four general practitioners. No clinical nor demographic information was presented in the survey; images were the only clues to come up with a conclusion. We obtained written informed consent from all physician participants.

All participants answered the evaluation images in identical order. The first set was a balanced image set, which does not affect human physicians, but may adversely affect the DL model’s performance due to the imbalanced training dataset. The second set was an imbalanced image set, which may favor DL models, even with aforementioned class imbalance mitigation strategies^15^, but also may be a practical measure for real-world clinical performance since the incidence represents real-world proportions of disease in a tertiary referral hospital.

Similar to the machine models, participants were requested to answer according to the same labeling priority order if more than one pathology was present in the image. Human raters were not aware of whether the test set was balanced or not.

The Severance Hospital Institutional Review Boards approved this study. (IRB No 2019–0467-001). All methods were performed in compliance with the Declaration of Helsinki.

### AI-HI cooperative classifier

Human physicians and DL models make classifications using different mechanisms; hence, combining both classifications would lead to improvement, similar to creating ensemble classifiers in DL models.

1. Evaluation of synergistic effects according to classes and human expertise.

Before introducing AI-HI cooperation models, we examined the synergistic effects of combining human diagnostic results and predictions by DL models according to human expertise and classes with different prevalences.

For the four best-performing DL model classifiers in the previous study^15^, a basic ensemble of the prediction results was done by adding each value following the softmax activation function. In mathematical notations,

$$ {\text{c}}^{*} = \mathop {\text{arg max}}\limits_{c} \left( {\mathop \sum \limits_{i = 1}^{n} \sigma \left( {M_{i} \left( x \right)} \right) + {{\alpha \delta }}\left( x \right)} \right),\;\; 0 \le {\upalpha } \le 4, $$

where ${\mathbf{\rm P}}_{{{\mathcal{M}}_{i} }} \left( x \right) = \sigma (M_{i} \left( x \right))$ generates a probability vector of being each class of the *i*-th model $M_{i}$ (among *n* = 4 models) for an input image *x* using a softmax function $\sigma$. ${\updelta }\left( x \right)$ is a function that takes the input from the human and returns a one-dimensional binarized vector, having 1 in the human-predicted class and 0 otherwise. α is a personalized human weight for adjusting the influence of the human-predicted class. If ${\upalpha }$ is set to 0, the human input is not used, whereas if ${\alpha  }$ is set to 4, the human input is always bigger than the sum of each four DL classifiers’ softmax values. ${\text{c}}^{*}$ is the final class prediction that has the maximal vector sum of all HI and DL classifiers. Upon inspection of maximum values in both test sets, they ranged from 0.9 to 3.65, with an average of around 2.5. This basic ensemble method corresponds to PoptMH, explained below.

We divided all participating human doctors into ENT and non-ENT groups. For each class, we evaluated the mean accuracies and the mean recalls for each class in the ENT group and non-ENT group according to different weights $\mathrm{\alpha }$. The recall of a class is defined by the number of true-positive samples divided by the number of true samples for the class.

The results are displayed in Fig. 1.

2. Basics of AI-HI cooperation models.

Considering individual differences in classification performance and quality, we proposed a cooperation strategy by appropriately weighing the classification results of both humans and DL models so that the classifier reflects the physician’s personal diagnostic preferences.

First, the system learns the human rater’s diagnostic characteristics by evaluating the class-specific bias and overall accuracy of the human rater from the expert’s diagnostic result of the evaluation dataset of a maximum of 300 balanced samples. Using a human expert’s confusion matrix of the evaluation dataset, we derived the conditional probability of the true class given each diagnostic decision (predicted label) for the human expert. Based on this, the optimal weight for the human expert compared to the DL classifiers is estimated. The mathematical formulation is explained below.

For an evaluation data set composed of N samples of images ${\varvec{X}}_{n = 1,..,N}^{E}$, let’s assume the i-th human rater ${\mathcal{H}}_{i}$ performs diagnostic decisions.

1. For *N* evaluation sample images $${\varvec{X}}_{n = 1,..,N}^{E}$$ for total *C* classes, we derive a set of a conditional probability vector $$\{ {\mathbf{\rm P}}_{{{\mathcal{H}}_{i} }}^{n} \}_{n = 1, \ldots ,N}$$,$$ {\mathbf{\rm P}}_{{{\mathcal{H}}_{i} }}^{n} \in R^{1 \times C}$$. $${\mathbf{\rm P}}_{{{\mathcal{H}}_{i} }}^{n}$$ is composed of a conditional probability $$p_{i} \left( {T_{l} {\text{|D}}_{n}^{i} } \right)$$ of being a true label $$T_{l} $$ given a diagnosed label $$ {\text{D}}_{n}^{i} $$ for the n-th image performed by the human rater $${\mathcal{H}}_{i}$$. The conditional probability $$p_{i} \left( {T_{l} {\text{|D}}_{n}^{i} } \right)$$ is calculated based on each individual’s confusion matrix $${\mathbf{C}}_{i}$$ (for the evaluation data set) by normalizing each column (predicted class) of the confusion matrix by the sum of the column along with the true label.
  $$ p_{i} \left( {T_{l} {\text{|D}}_{n}^{i} } \right) = \frac{{{\mathbf{C}}_{i} \left( {T_{l} ,{\text{D}}_{n}^{i} } \right)}}{{\mathop \sum \nolimits_{k = 1}^{C} {\mathbf{C}}_{i} \left( {T_{k} ,{\text{D}}_{n}^{i} } \right)}} $$

Thus, a conditional probability vector for a sample *n* is defined by

$${\mathbf{\rm P}}_{{{\mathcal{H}}_{i} }}^{n} = \left[ {p_{i} \left( {T_{1} {\text{|D}}_{n}^{i} } \right) p_{i} \left( {T_{2} {\text{|D}}_{n}^{i} } \right) \cdots p_{i} \left( {T_{C} {\text{|D}}_{n}^{i} } \right)} \right]$$.

Each expert has a matrix of conditional probabilities for all evaluation samples.

$$ {\mathbf{\rm P}}_{{{\mathcal{H}}_{i} }} = \left[ {{\mathbf{\rm P}}_{{{\mathcal{H}}_{i} }}^{1} \ldots {\mathbf{\rm P}}_{{{\mathcal{H}}_{i} }}^{N} } \right]^{T} \in R^{N \times C} $$

2. For the same ${\varvec{X}}_{n = 1,..,N}^{E}$, we calculate a classification probability matrix (the output of the softmax layer) for each DL model, e.g., for j-th model ${\mathcal{M}}_{j}$, ${\mathbf{\rm P}}_{{M_{j} }} = \left[ {{\mathbf{\rm P}}_{{{\mathcal{M}}_{j} }}^{1} \ldots {\mathbf{\rm P}}_{{{\mathcal{M}}_{j} }}^{N} } \right]^{T} \in R^{N \times C}$. We then concatenate all DL and the i-th human rater ${\mathcal{H}}_{i}$’s probability matrices, i.e., $\left[ {{\mathbf{\rm P}}_{{{\mathcal{M}}_{1} }} \ldots {\mathbf{\rm P}}_{{{\mathcal{M}}_{M} }} {\mathbf{\rm P}}_{{{\mathcal{H}}_{i} }} } \right] \in R^{{N \times C \times \left( {M + 1} \right) }}$ to derive optimal weights for classes for the human expert.
3. The optimal weights for a total of *M* classifiers and a human rater, ${\mathbf{W}}^{*} \in R^{{\left( {M + 1} \right) \times C}} ,$ are determined to best fit the target ground-truth labels ${\mathbf{Y}}$ for the evaluation dataset (for each sample *n,* the n-th row of ${\mathbf{Y}}$ is assigned with 1 for the sample label, otherwise all zeros, thus ${\mathbf{Y}} \in R^{N \times C} )$ in terms of cross-entropy (CE) loss between weighted sum of diagnosis $\left( {\left[ {{\mathbf{\rm P}}_{{{\mathcal{M}}_{1} }} \ldots {\mathbf{\rm P}}_{{{\mathcal{M}}_{M} }} {\mathbf{\rm P}}_{{{\mathcal{H}}_{i} }} } \right]{\varvec{W}} \in R^{N \times C } } \right)$ and ${\mathbf{Y}}$.
  $$ {\mathbf{W}}^{*} = arg\mathop {\min }\limits_{{\mathbf{W}}} CE\left( {{\mathbf{Y}},\left[ {{\mathbf{\rm P}}_{{{\mathcal{M}}_{1} }} \ldots {\mathbf{\rm P}}_{{{\mathcal{M}}_{M} }} {\mathbf{\rm P}}_{{{\mathcal{H}}_{i} }} } \right]{\varvec{W}}} \right) $$
4. For the final procedure for a new test sample, ${\varvec{X}}^{{\varvec{t}}}$, the system choose the class of maximum probability (argmax) from the weighted probabilities drawn from DL models and the human rater.
  $$ C_{i}^{*} = arg\mathop {\max }\limits_{c} \left[ {{\text{\rm P}}_{{{\mathcal{M}}_{1} }}^{t} \ldots {\text{\rm P}}_{{{\mathcal{M}}_{M} }}^{t} {\text{\rm P}}_{{{\mathcal{H}}_{i} }}^{t} } \right]{\mathbf{W}}^{*} $$

**Figure 4:**
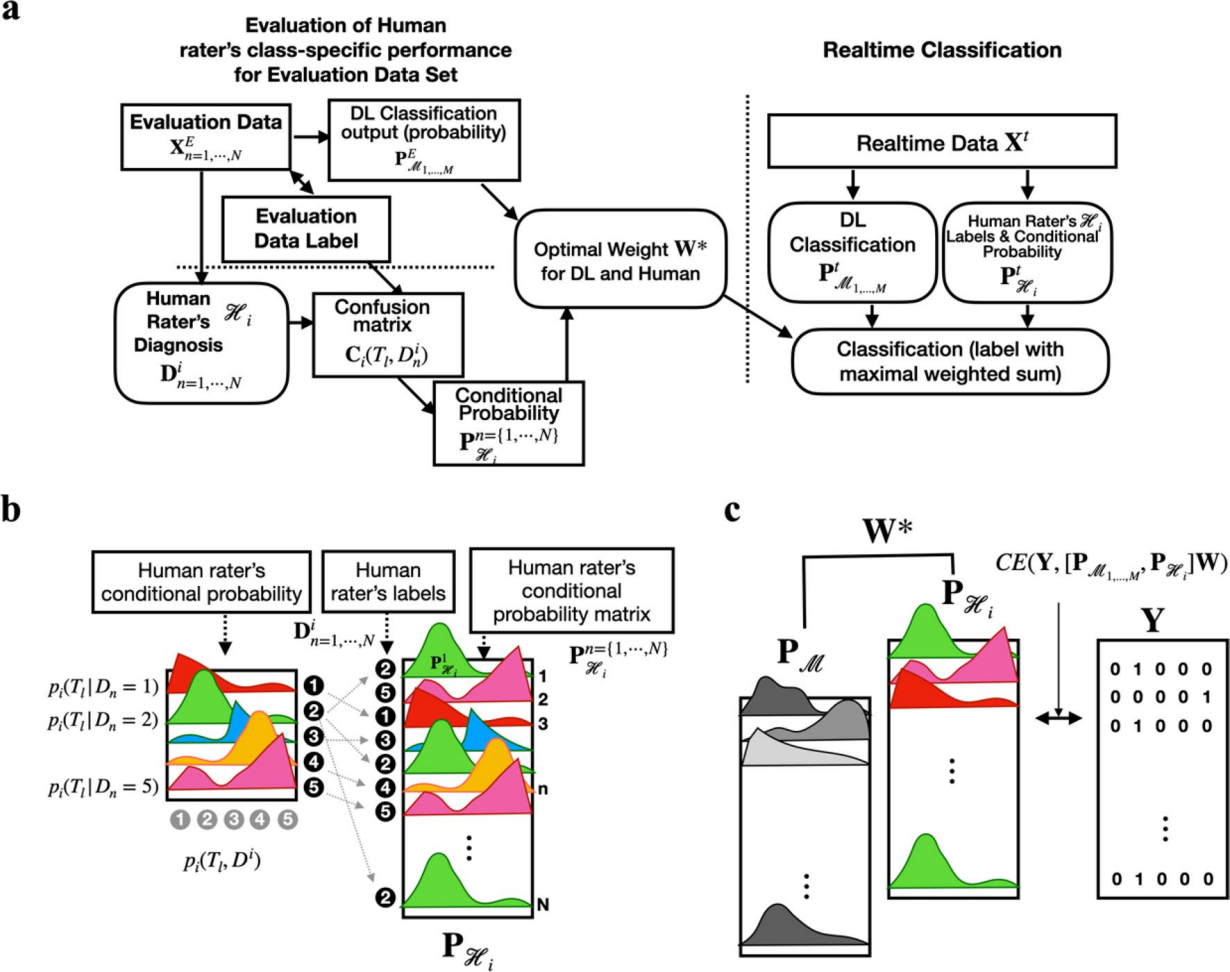
The procedure for the human–machine cooperation model. (**a**) The evaluation procedure of human rater’s performance and the real-time application of the cooperation model. (**b**) The procedure to derive the conditional probability of each human rater from the confusion matrix of the evaluation data set was explained. The conditional probability matrix derived from the confusion matrix of the evaluation data set for each individual was assigned to each sample according to the human rater’s diagnosis for the sample. (**c**) Optimal weight was determined by minimizing conditional entropy (CE) between the label for the evaluation data set and the weighted sum of human and machine probability matrix.

Figure 4 illustrates the current procedure.

3. Evaluations of AI-HI cooperation models in human individuals according to evaluation sample sizes.

To utilize small data samples for the evaluation of each individual and to reduce the number of parameters to estimate, we simply averaged the classification results of four machine models as they show a more or less similar pattern of classification. We then combined the classification result of one machine model and one human rater by weighting them with a single ratio variable W = {*w*}, rather than a vector.

For a human expert ${\mathcal{H}}_{i}$, the human diagnostic decision for a new test data, ${\varvec{X}}_{{}}^{t}$, is denoted as delta-response vector $\delta_{{{\mathcal{H}}_{i} }}^{t}$(assigning only the chosen label to be one, others to be zeros). The conditional probability (${\text{\rm P}}_{{{\mathcal{H}}_{i} }}^{t} )$ of the true label given a human diagnosed label is derived from the delta-response. The weighted sum $\left( {{\text{S}}_{i}^{t} } \right)$ of the classification probability of the DL classifiers (${\text{\rm P}}_{{\mathcal{M}}}^{t} )$ and the conditional probability of the human decision (${\text{\rm P}}_{{{\mathcal{H}}_{i} }}^{t} )$ is used to classify new samples. The three different cooperation models are based on whether the conditional probability is used and whether the weight for the human and machine’s decision is optimized based on the human individual’s overall accuracy.

$$ {\text{S}}_{i}^{t} = 0.5{\text{ {\rm P}}}_{{\mathcal{M}}}^{t} + 0.5{\text{ {\rm P}}}_{{{\mathcal{H}}_{i} }}^{t} \;\;\left( {{\text{CavgMH}}} \right) $$

$$ {\text{S}}_{i}^{t} = \left( {1 - w_{i}^{*} } \right){\text{\rm P}}_{{\mathcal{M}}}^{t} + w_{i}^{*} \delta_{{{\mathcal{H}}_{i} }}^{t} \;\;\left( {{\text{PoptMH}}} \right) $$

$$ {\text{S}}_{i}^{t} = \left( {1 - w_{i}^{*} } \right){\text{\rm P}}_{{\mathcal{M}}}^{t} + w_{i}^{*} {\text{\rm P}}_{{{\mathcal{H}}_{i} }}^{t} \;\;\left( {{\text{PCoptMH}}} \right) $$

From the evaluation data set conducted by a human expert ${\mathcal{H}}_{i}$, the optimal weight for the human $w_{i}^{*}$ is estimated for PoptMH and PCoptMH by minimizing the cross-entropy loss between ${\text{S}}_{i}^{E}$ of the evaluation data set and ${\text{Y}}^{E}$, the true label for the evaluation data set.

For all three different models, the final label was chosen to maximize ${\text{S}}_{i}^{t}$ for each individual.

$$ C_{i}^{{\text{t*}}} = arg\mathop {\max {\text{S}}_{i}^{t} }\limits_{c} $$

To test how many evaluation samples are needed to reliably assess the diagnostic characteristics of each individual, the system evaluates each human expert using a portion of 300 balanced data, i.e., 30 (6 samples per class), 60 (12 samples per class), 100 (20 samples per class), and 150 (30 samples per class) evaluation samples. The average accuracies of the five-fold training and tests were used to evaluate the performance of the cooperations models in learning the individual and choosing the minimal number of evaluation samples.

## Data Availability

The data that support the findings of this study are available from the corresponding author upon reasonable request.
